# DNA cytosine methylation dynamics and functional roles in horticultural crops

**DOI:** 10.1093/hr/uhad170

**Published:** 2023-08-29

**Authors:** Peipei Liu, Ruie Liu, Yaping Xu, Caixi Zhang, Qingfeng Niu, Zhaobo Lang

**Affiliations:** National Engineering Laboratory of Crop Stress Resistance Breeding, School of Life Sciences, Anhui Agricultural University, Hefei, 230036, China; School of Agriculture and Biology, Shanghai Jiao Tong University, Shanghai 200240, China; Shanghai Center for Plant Stress Biology, National Key Laboratory of Plant Molecular Genetics, Center for Excellence in Molecular Plant Sciences, Chinese Academy of Sciences, Shanghai 201602, China; School of Agriculture and Biology, Shanghai Jiao Tong University, Shanghai 200240, China; National Engineering Laboratory of Crop Stress Resistance Breeding, School of Life Sciences, Anhui Agricultural University, Hefei, 230036, China; Institute of Advanced Biotechnology, School of Life Sciences, Southern University of Science and Technology, Shenzhen 518055, China

## Abstract

Methylation of cytosine is a conserved epigenetic modification that maintains the dynamic balance of methylation in plants under the regulation of methyltransferases and demethylases. In recent years, the study of DNA methylation in regulating the growth and development of plants and animals has become a key area of research. This review describes the regulatory mechanisms of DNA cytosine methylation in plants. It summarizes studies on epigenetic modifications of DNA methylation in fruit ripening, development, senescence, plant height, organ size, and under biotic and abiotic stresses in horticultural crops. The review provides a theoretical basis for understanding the mechanisms of DNA methylation and their relevance to breeding, genetic improvement, research, innovation, and exploitation of new cultivars of horticultural crops.

## 1. Introduction

Epigenetic changes include cytosine methylation, non-coding RNAs, RNA modifications, histone modifications, and chromatin remodeling, which can interact and determine the specific state of chromatin, thereby repressing or activating gene expression [1, 2]. Cytosine methylation is among the better understood mechanisms in epigenetics that effectively regulate genome stability. Cytosine methylation modifies the fifth carbon atom of cytosine and is a conserved epigenetic change that plays vital roles in plants and eukaryotes [3, 4]. In plants, cytosine methylation occurs at symmetric cytosine sequences (CG or CHG) and asymmetric cytosine sequences (CHH), maintaining a dynamic balance in total genomic methylation under the combined action of methyltransferase and demethylase [3, 5]. Three main classes of methyltransferases are known in plants: domain-rearranged methyltransferases (DRMs), DNA methyltransferase 1 (MET1), and chromomethylase 3 (CMT3). The methylation status of cytosine is established or maintained by these specific methyltransferases [6–8]. The active DNA demethylation pathway is initiated by a family of bifunctional 5-methylcytosine (5mC) DNA glycosylases–apurinic/apyrimidinic lyases through base excision repair in plants [9, 10]. In *Arabidopsis*, there are four demethylases, DEMETER (DME), AtDML2, and AtDML3, and REPRESSOR OF SILENCING 1 (AtROS1), all of which can excise 5mC [5, 11].

DNA methylation is a conserved epigenetic modification that serves a crucial function in various tissues and cells during plant growth and development, including fruit plants. Fruits are organs unique to angiosperms that facilitate seed dispersal and are an important nutritional food source for humankind. The two main types of fruits are dry fruits [e.g. walnut (*Juglans regia*)] and fleshy fruits [e.g. apple (*Malus* × *domestica*)] [1]. Fruit development can be divided into three stages: fruit cell division and expansion, and fruit ripening [12]. Based on the fruit ripening process, fleshy fruits can be further classified into climacteric fruits [e.g. kiwifruit (*Actinidia deliciosa*) and tomato (*Solanum lycopersicum*)] and non-climacteric fruits [e.g. citrus (*Citrus* spp.) and strawberry (*Fragaria* × *ananassa*)]. The ripening process of climacteric fruits is accompanied by increased ethylene production and a respiratory burst, whereas the development of non-climacteric fruits lacks respiratory and ethylene-release peaks.

Although previous epigenetic studies were mainly limited to model species such as *Arabidopsis thaliana*, the recent increase in number of sequenced and assembled genomes of horticultural crops and new technological developments have facilitated studies of epigenetic changes in horticultural plants, including vegetable and fruit crops. Tomato is an economically important vegetable and a model plant for studying the ripening of fleshy fruits. Fruit development and ripening involves various biochemical and physiological changes, such as in pigments, flavor volatiles, and soluble sugar accumulation [13]. Phytohormonal and developmental factors drive these changes. Among studies of the epigenetic control of fruit ripening and development, tomato has been a frequent subject because it can be genetically modified, has a well-assembled genome, and is a source of many mutants with developmental defects.

Unlike animals, which can actively and spontaneously avoid adverse environments, plants are frequently subject to natural biotic and abiotic stresses. Stable DNA methylation levels are required for normal plant development; excessively high or low methylation levels can lead to abnormal plant growth and morphological defects [14]. Plants are faced with different biotic and abiotic stresses that lead to increased gene methylation and reduced genomic activity. Biotic stresses mainly comprise the stresses on plant growth and development caused by interactions with viruses, pathogens, and pests, mediating changes in DNA cytosine methylation patterns and levels in response.

In this review, we first summarize the patterns of DNA cytosine methylation in plants and the mechanism of cytosine methylation in transcriptional regulation. In addition, we review the crucial roles of DNA cytosine methylation dynamics in horticultural plants, including their roles in regulating plant growth and fruit development and ripening, and biotic and abiotic stress responses. We emphasize recent discoveries that have helped to provide an improved understanding of the regulation and function of DNA cytosine methylation in horticultural plants.

## 2. Patterns of DNA cytosine methylation in plants

In plants, DNA cytosine methylation patterns are formed through three processes: methylation maintenance, *de novo* DNA methylation, and active DNA demethylation.

### 2.1. *De novo* methylation

**Figure 1:**
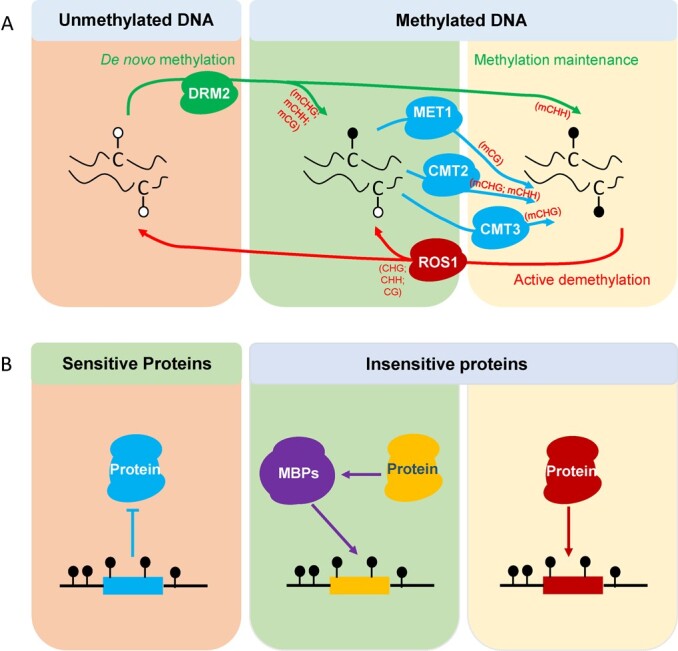
Dynamic regulation of cytosine methylation in plants. (A) In plants, cytosine methylation modifications arise mainly in the context sequences (CG, CHG, and CHH) and are catalyzed by DNA methyltransferases with different regulatory mechanisms; CG-type methylation is maintained by MET1, which recognizes the mCG context in parental DNA and methylates the unmethylated cytosine in the daughter strand during DNA replication. CMTs, plant-specific cytosine methyltransferases, maintain the CHG and CHH methylation. *Arabidopsis* CMT3 maintains CHG-type methylation and CMT2, to a much lesser extent, mediates CHG methylation. DRM2 maintains CHH methylation in the RNA-directed DNA methylation (RdDM) target region via the RdDM pathway. *De novo* methylation uses the RdDM pathway to guide DRM2 in the establishment of cytosine methylation. Active DNA demethylation is an enzymatic reaction involving the ROS1 family of glucosylases in plants. (B) Proteins sensitive to cytosine methylation (some transcription factors) are repressed from binding by mCGs, mCHH, or mCHG within their motifs, causing steric hindrance or alteration of the DNA shape. Methyl-binding domain proteins (MBPs: MBD domain proteins or SRA domain proteins) recognize mCGs, mCHH, or mCHG in a sequence-independent manner. Transcription factors can bind to the DNA sequence containing mCGs, mCHH, or mCHG through direct interaction with MBPs. DNA methylation-insensitive proteins will bind their motifs regardless of the surrounding DNA methylation state (e.g. LEAFY). Insensitive proteins bind to the DNA sequence motifs containing methylcytosine through direct affinity.

In plants, *de novo* methylation uses the RNA-directed DNA methylation (RdDM) pathway to guide DRM2 in the establishment of cytosine methylation. The RdDM pathway, which involves scaffold RNAs, small interfering RNAs (siRNAs), and a variety of RdDM components, is a plant-specific pathway [4]. In the RdDM pathway, the plant-specific RNA polymerase IV (Pol IV), which evolved from Pol II, plays a crucial role in initiating *de novo* DNA methylation. Pol IV generates single-stranded RNAs (ssRNAs). The ssRNAs are subsequently used as a template to synthesize double-stranded RNA (dsRNA) mediated by RNA-DEPENDENT RNA POLYMERASE 2 (RDR2). The dsRNA is then cut to generate 24-nucleotide siRNAs by DICER-LIKE 3 (DCL3) [15]. The siRNAs are stabilized by HUA ENHANCER 1 (HEN1) through methylation at their 3′-OH groups [16], and are subsequently loaded on ARGONAUTE 4 (AGO4) or AGO6. POL V transcripts are considered to pair with the siRNAs and recruit the other suppressors mediating *de novo* methylation by DRM2 (Fig. 1A).

Thus, in the RdDM pathway, cytosine methylation loci are determined and modified by the sites targeted by Pol IV, Pol V, and DRM2. The protein SAWADEE HOMEODOMAIN HOMOLOG 1 (SHH1) can recruit Pol IV to the chromatin regions packed with H3K9me2 [17]. In addition, SHH2 [18] in maize (*Zea mays*) and the Pol V-binding protein RDM15 [19] in *Arabidopsis* specifically recognize and bind to H3K9me1 and H3K4me1, respectively, indicating that the diverse histone modifications to methylated DNA-containing loci influence the function of Pol V. In addition to the production of 24-nt siRNAs by Pol IV–RDR2–DCL3, there are some other small RNA pathways that can direct RdDM and are known as non-canonical RdDM mechanisms [20].

### 2.2. Methylation maintenance

Maintenance of methylation refers to the occurrence of cytosine methylation on the nascent strand of the DNA duplex where methylation occurs. In plants, DNA methylation modifications arise mainly in the CG, CHG, and CHH sequences, and are catalyzed by DNA methyltransferases with different regulatory mechanisms; CG-type methylation is maintained by MET1, which recognizes the methylated CG context in parental DNA and methylates the unmethylated cytosine in the daughter strand during DNA replication [21]. CHROMOMETHYLASE (CMT), a plant-specific DNA methyltransferase, maintains CHG and CHH methylation [22]. The *Arabidopsis* genome contains three *CMT* genes [23], among which *CMT3* maintains CHG-type methylation and *CMT2*, to a much lesser extent, mediates CHG methylation [24]. In addition, H3K9me2 modifications and methylated CHG may strengthen each other through regulatory feedback loops [25]. Asymmetric CHH methylation is maintained by CMT2 and DRM2 methyltransferases (Fig. 1A) [26].

### 2.3. DNA demethylation

Cytosine methylation is a reversible epigenetic modification. The cytosine methylation level is controlled by demethylation and methylation pathways, and DNA demethylation can activate genes that are in a silent state [17, 27]. Cytosine demethylation can be categorized into an active demethylation pathway and a passive demethylation pathway. Active demethylation is catalyzed by DNA demethylation enzymes and is not dependent on DNA replication. In plants, active cytosine demethylation is an enzymatic reaction involving the ROS1 family of glucosylases (Fig. 1A) [28]. Unlike mammals, plants directly excise the 5mC base utilizing 5mC DNA glycosylases [29]. ROS1, AtDME, AtDML2, and AtDML3, which are members of the ROS1 family, are responsible for the demethylation of different tissues in somatic cells [30]. In *Arabidopsis*, METHYL-CPG-BINDING DOMAIN PROTEIN 7 (MBD7) can recruit INCREASED DNA METHYLATION 1 (IDM1) through physical interaction with IDM2 and IDM3. These proteins form a complex that creates a suitable chromatin state to recruit ROS1 and therefore regulate DNA demethylation [11]. Recently, the chromatin-remodeling complex SWR1 was newly reported to be a component required for ROS1-mediated DNA demethylation [11]. Passive demethylation is a process in which DNA methylation levels are reduced owing to inactivation or inhibition of DNA methylation transferase during DNA replication [4, 31].

## 3. Mechanism of DNA cytosine methylation in transcriptional regulation

DNA cytosine methylation plays an important role in transcriptional regulation and, therefore, is involved in the control of many biological processes. Disruption of DNA methylation in plants can lead to growth and developmental disorders, such as during fertilization, seed development, and fruit development [3, 32, 33]. In eukaryotes, DNA methylation plays a dual role of silencing genes and transposable elements (TEs) as well as stimulating gene transcription. For instance, two CG-specific DNA methylation readers, MBD5 and MBD6, were found to redundantly repress methylated genes and transposons in *A. thaliana* by recruiting the J-domain protein SILENZIO [34]. Additionally, DNA methylation can also activate gene expression. Two SU(VAR)3-9 homologs were identified in *Arabidopsis*, and the transcriptional anti-silencing proteins SUVH1 and SUVH3 were found to bind to methylated DNA as methyl readers. Furthermore, two DNAJ domain-containing homologs, DNAJ1 and DNAJ2, were recruited to form complexes that enhance proximal gene expression [35]. However, only ~5% of genes are methylated in the promoter region in *Arabidopsis* [36]. Consequently, the activation of a small proportion of genes is affected by DNA methylation. Conversely, horticultural crops with larger genomes have substantially more genes with methylated promoters [37, 38]. Therefore, cytosine methylation dynamics and mutants of crops are ideal materials to study the molecular functions of cytosine methylation in the control of gene expression. DNA hypermethylation of promoters usually represses gene activation, although in some cases it promotes gene activation; for example, hundreds of ripening repressors maintain higher expression levels in the immature stage than in the ripening stage [38].

Cytosine methylation and RIPENING INHIBITOR (RIN) binding capacity have been analyzed during fruit ripening in the wild type (WT) and *Cnr* and *rin* mutants in tomato. The results show that RIN fails to bind to the binding sites in hypermethylated *Cnr* mutants, indicating that cytosine methylation changes might contribute to the control of gene transcription through promoter activity [37, 39]. Liu *et al*. demonstrated active demethylation in the promoter of *RIN*, *PHYTOENE SYNTHASE 1* (*PSY1*), and *NON-RIPENING* (*NOR*), the activation of which is necessary for fruit ripening [40]. Lang *et al*. generated whole-genome cytosine methylation and the transcriptome for WT and *sldml2* mutant fruit at the immature and ripening stages [38]. The results showed that active cytosine demethylation in the promoter is required for activation of hundreds of genes involved in ripening, such as *RIN* and *POLYGALACTURONASE 2A* (*PG2A*). Surprisingly, the authors reported that active cytosine demethylation in the promoter is required for repression of hundreds of ripening-repressed genes, such as *CHLOROPHYLL A-B BINDING PROTEIN CP24 10B* (*SlCAP10B*) and *RUBISCO SMALL UNIT 2A* (*SlRBCS-2A*) [38]. However, the mechanisms underlying the silencing role of DNA demethylation remain unknown. In the skin of pear and apple fruit, cytosine methylation in the *MYB10* promoter causes gene silencing and decreased anthocyanin accumulation in the fruit [41, 42].

Most transcription factors (TFs) are inhibited by DNA methylation because the 5-methyl group of methylcytosine often clashes with protein residues involved in specific base pairing [43]. By contrast, some TFs are insensitive or favor a methylcytosine motif, possibly because hydrophobic phases are formed under interaction between the methylated motif and the TF proteins (Fig. 1B). Homeodomain TFs, some basic leucine zipper TFs, and some pioneer TFs [43], such as *LEAFY*, have been reported to belong to this group [44]. There is a possibility that cytosine methylation controls gene activities through enhancing the binding capability of some transcription activators or may inhibit the binding capability of some transcription repressors (Fig. 1B). Cistrome and epicistrome maps have proved effective for elucidation of complex transcriptional networks in *Arabidopsis*, maize, and wheat (*Triticum aestivum*) [45, 46]. However, such reports on horticultural crops are lacking. Methyl-CpG binding proteins (MBDs) are a class of methylcytosine-binding domain proteins that affect gene transcription through recruitment activators or repressors in *Arabidopsis* (Fig. 1B) [47]. In tomato, SlMBD5 is a specific meCpG-binding protein that enhances transcription activity in the regulation of pigmentation and has pleiotropic effects on plant development [48]. However, how cytosine methylation in the promoter activates gene transcription *in vivo* is still poorly understood.

## 4. DNA cytosine methylation in plant growth and development

DNA cytosine methylation is an essential epigenetic modification involved in many biological processes. The levels of cytosine methylation in different tissues are tightly regulated during plant growth and throughout the life cycle, reflecting the critical role of cytosine methylation in plant biology. Published studies indicate that epigenetic engineering holds immense potential in modern plant breeding, particularly in the species with limited genetic diversity (Table 1).

**Table 1:** Biological processes involving DNA methylation dynamics in horticultural crops.

**Species**	**Mechanism**	**Biological function**	**References**
Tomato (*Solanum lycopersicum*)	SIDML2-mediated whole-genome DNA demethylation	Fruit ripening	[38, 40]
	SlMET1-mediated whole-genome DNA methylation	Leaf and inflorescence development	[49]
	Plastid whole-genome DNA methylation	Chromoplasts	[50]
	Chilling-induced DNA hypermethylation	Loss of flavor	[51]
	Expression of Pi starvation response genes is associated with DNA methylation	Phosphate starvation (Pi−)	[52]
	DNA hypermethylation-enhanced virus gene transcriptional silencing	Geminivirus resistance	[53]
Strawberry (*Fragaria vesca*)	Whole-genome DNA demethylation mediated by RdDM components	Fruit ripening	[12]
	Whole-genome DNA demethylation mediated by FDM1	Plant height and organ size	[54]
Apple (*Malus*)	Whole-genome cytosine hypomethylation is critical for rejuvenation-dependent adventitious rooting ability	Adventitious root growth	[55]
	Hypomethylation in the promoter of *MYB10*	Anthocyanin synthesis	[42]
	High expression of flowering-related genes in spiny buds is associated with low methylation levels in genomic regions	Bud formation	[56]
	Chilling-induced whole-genome DNA demethylation	Chilling stress	[57]
Orange (*Citrus sinensis*)	Global increase in cytosine methylation in fruit ripening	Fruit ripening	[3]
Grape (*Vitis vinifera*)	Melatonin treatment broadly decreases methylation levels	Disease resistance and flavonoid biosynthesis	[58]
Peach (*Prunus persica*)	Temperature-dependent whole-genome DNA demethylation	Anthocyanin biosynthesis	[59]
	Hypomethylation in the promoter of *PpTPS3*	Plant terpene biosynthesis	[60]
	Whole-genome DNA hypomethylation	Callus formation	[61]
Pear (*Pyrus pyrifolia*)	Dynamic of whole-genome DNA methylation in debagged and bagged treatments	Anthocyanin biosynthesis	[62]
Cucumber (*Cucumis sativus*)	Dynamic of whole-genome DNA methylation in the apex	Temperature-dependent sex determination	[63]
	Demethylation of ribosomal RNA genes is induced by hop stunt viroid infection	Gametophyte development	[64]
Melon (*Cucumis melo*)	DEGS is hypermethylated under *P. xanthii* stress	DNA methylation is involved in resistance to *P. xanthii*	[65]
Potato (*Solanum tuberosum*)	Genome-wide demethylation in response to *Fusarium* toxin deoxynivalenol	Potato disease resistance	[66]
Cabbage (*Brassica rapa*)	DNA methyltransferase decreased	Leaf senescence	[67]
Pepper (*Capsicum annuum*)	Global decrease in cytosine methylation	Fruit ripening	[68]
Chrysanthemum (*Chrysanthemum* × *morifolium*)	Hypomethylation occurs during floral development	Floral transition	[69]
Feng Dan (*Paeonia ostii*)	Whole-genome DNA demethylation occurs during seed development	Seed development	[70]
Tea plant (*Camellia sinensis*)	DNA demethylase upregulated and DNA methylase repressed during withering processing	Tea flavor	[71]
	Chilling induced whole-genome DNA demethylation	Gene duplication and chilling tolerance	[72]
Mulberry (*Morus notabilis*)	Transposable element and gene hypomethylation occur in resistance to *B. cinerea*	Pathogenic responses and resistance	[73]
Willow (*Salix viminalis*)	Whole-genome DNA hypomethylation stimulates during flower development	Plant reproductive transition and early floral development	[74]
Oil palm (*Elaeis guineensis*)	Whole-genome DNA hypermethylation in embryogenic suspensions	Embryogenic capacities	[75]

### 4.1. Fruit development and ripening

The pioneering work of Hadfield *et al*. examined the reduced methylation levels of two ripening-specific genes, *polygalacturonase* (*PG*) and *cellulase* (*CEL*), during fruit ripening [76]. The isoschizomer pair *Hpa*II/*Msp*I, which show differential sensitivity to 5mC at the restriction site, was applied to infer the cytosine methylation status. Southern blot analysis revealed cytosine demethylation in sequences containing the *cellulase* or *polygalacturonase* genes during fruit ripening [76]. The control of fruit ripening may be facilitated by cytosine demethylation. Early evidence indicated that fruit ripening is associated with a global decrease in cytosine methylation in plants [77]. Whole-genome bisulfite sequencing in fruit at different developmental stages further confirmed the genome-wide DNA demethylation during fruit ripening in tomato [39]. Demethylation is likely to be involved in the epigenetic control of fruit ripening [37]. Recently, Lang *et al*. and Liu *et al*. demonstrated that active cytosine demethylation is necessary for tomato fruit ripening and there is a direct causal relationship between active DNA demethylation and induction of ripening-related gene expression in tomato fruit [38, 40]. In addition, the tomato DNA demethylase SlDML2, an ortholog of AtROS1 in *Arabidopsis*, mediates active cytosine demethylation at the onset of fruit ripening. Comparing the methylation landscape and transcriptome in fruit of the WT and *sldml2* mutant showed that dramatically increased *SlDML2* expression is required for global cytosine demethylation at the onset of fruit ripening. In addition to the active cytosine demethylation pathway, the components of the RdDM pathway have been shown to play critical roles in plant growth and fruit development in tomato [40].

Evidence for the role of cytosine methylation in controlling tomato fruit development and ripening is convincing, but determining whether the mechanisms identified in tomato are conserved requires their investigation in additional fruit species. Cheng *et al*. generated single-base-resolution profiles of the DNA methylome of immature and mature non-climacteric fruit of strawberry. The results showed comprehensive decreased cytosine methylation levels during fruit ripening [12]. These results suggested that ripening-induced DNA hypomethylation occurs in both non-climacteric fruit (e.g. tomato) and climacteric fruit (e.g. strawberry). In contrast to the mechanism of DNA hypomethylation during tomato ripening, no DNA demethylase genes were upregulated during strawberry fruit ripening. Interestingly, genes involved in RdDM were downregulated, and ripening-induced cytosine hypomethylation was associated with reduced siRNA levels, which coincides with reduced methylation levels. Thus, the downregulation of RdDM contributes to passive cytosine demethylation during fruit ripening in strawberry. In addition, application of DNA methylation inhibitors resulted in an early-ripening phenotype in strawberry fruit, suggesting that DNA hypomethylation is essential for initiation of fruit ripening in strawberry. Unlike the dynamics of DNA methylation in tomato or strawberry, global cytosine methylation increases during fruit ripening in sweet orange (*Citrus sinensis*) [3]. Whole-genome bisulfite sequencing (WGBS) and transcriptome analysis revealed DNA hypermethylation consistent with the expression of DNA demethylase genes repression in sweet orange. The DNA hypomethylation also occurred in the promoter region of ripening-related genes during the transition period in pepper (*Capsicum annuum*) fruit owing to upregulation of *DML2-like* and downregulation of *MET1-like 1*, *MET1-like 2*, *CMT2-like*, and *CMT4-like* [68]. The epigenome dynamics have also been profiled in apple, banana, melon (*Cucumis melo*), papaya (*Carica papaya*), peach (*Prunus persica*), pear, cucumber (*Cucumis sativus*), grape (*Vitis vinifera*), and watermelon (*Citrullus lanatus*) [78]. The results also showed the diversity of cytosine methylation changes during fruit development and ripening, and confirmed the observation that DNA methylation dynamics vary between species. DNA methylation changes are involved not only in the control of ripening but also in the regulation of fruit size and metabolite accumulation in fruit [79]. In apple, anthocyanin accumulation in the fruit is negatively correlated with the cytosine methylation levels at the *MdMYB10* promoter, and whole-genome CHH methylation is correlated with fruit size (Table 1) [42]. It is apparent, therefore, that plant species dynamically regulate DNA methylation to regulate fruit growth and ripening, but this may vary between species.

### 4.2. Vegetative and reproductive development

Among tomato mutants defective in RdDM pathway components, *slnrpd1* mutants are defective in leaf, flower, and fruit development, *slnrpe1* mutants are infertile [80], while the *slmet1* mutant shows defective leaf and inflorescence development [49]. These studies suggest that the DNA methylation pathway proteins play a crucial role in vegetative and reproductive growth in tomato. DNA methylation also influences flower bud formation in apple. Through the analysis of DNA methylation changes and transcriptional responses in flower buds with varying flowering abilities, it was discovered that genes related to flowering and transcription factors were more prevalent in spur buds with the highest flowering rate. This was found to be correlated with lower levels of methylation in genomic regions [56]. In strawberry (*Fragaria vesca*), the *ros* mutant shows reduction in the size of the leaves, flowers, and fruits, and harbors a mutation in Factor of DNA Methylation 1 (FDM1). WGBS revealed that DNA methylation was remarkably reduced in *fvefdm1* knockout mutants. Further study revealed that FveFDM1 contributes to plant growth and development through DNA methylation mediated by RdDM [54]. In addition, DNA methylation may be associated with bud dormancy release. DNA methylation was increased in sweet cherry (*Prunus avium*) under chilling accumulation, and hypermethylation of two dormancy-related genes, *PavMADS1* (*PavDAM3*) and *PavMADS3* (*PavDAM5*), is associated with decreased expression levels [81]. Leaf senescence is influenced by various internal factors and the external environment, among which DNA modification is an essential factor [82]. Recent studies have reported that knockdown of *DML3* in *Arabidopsis* increases genomic DNA methylation levels, thereby suppressing the expression of plant senescence-related genes and delaying leaf senescence [83]. In horticultural crops, the epigenetic changes involved in the control of plant leaf senescence are largely unknown. Li *et al*. reported that postharvest senescence in cabbage (*Brassica rapa*) might be caused by cytosine demethylation of plant senescence-associated genes owing to the downregulation of DNA methyltransferases (BcCMT3, BcMET1, and BcDRM2) [67]. According to recent studies, DNA methylation is involved in callus formation and shoot regeneration in horticultural crops. In peach, DNA hypomethylation can activate auxin- and cytokinin-related regulators, leading to callus formation and shoot regeneration [61]. Similarly, DNA methylation affects the ability to form calli and regenerate shoots in strawberry, which influences the expression of some regeneration factors [84]. These studies revealed the potential functional roles of DNA methylation in regulating vegetative development.

Similarly, DNA methylation also plays an essential role in reproductive development. Chrysanthemum (*Chrysanthemum* × *morifolium*) is an important ornamental plant and a typical short-day plant. Methylation-sensitive amplification polymorphism (MSAP) and high-performance liquid chromatography (HPLC) have been used to examine the changes of DNA methylation during head bud emergence in two varieties of chrysanthemums that are early-flowering and late-flowering, respectively. The results showed that the level of methylation decreased more in the early-flowering variety than in the late-flowering variety during flower development [69]. *Paeonia ostii* ‘Feng Dan’ is an important woody oil plant in China, and its seed development is a complex process in which epigenetic regulation plays a crucial role. To understand the epigenetic regulation during seed development, the seed methylation level and pattern of *P. ostii* ‘Feng Dan’ were analyzed. Demethylation occurred mainly during seed development and was concentrated at the CG locus. Analysis of the homology of differentially methylated fragments revealed that they were mainly associated with metabolism, transcription, signal transduction, gene expression, and development. This suggests that seed development in *P. ostii* ‘Feng Dan’ may be regulated by DNA methylation, providing a valuable basis for further studies on the molecular mechanisms of seed development. Cheng *et al*. suggest that the initiation of flowering in *Salix viminalis* and subsequent floral growth are promoted by hypomethylation in leaves during the flowering transition stage [74].

## 5. Cytosine methylation alternation under biotic and abiotic stresses

### 5.1. Roles of cytosine methylation in response to biotic stress

Biotic stress can activate the plant immune system, including recognizing pathogen-related molecular patterns and activating basic defense systems, thus triggering specific defense mechanisms. Generally, DNA hypomethylation occurs when a plant experiences pathogen attack [85]. For example, in *Arabidopsis* the transcription of immune response genes and some defense genes are activated through transposon DNA demethylation, thereby limiting the proliferation of the bacterial pathogen *Pseudomonas syringae* in leaves and its invasion of vascular bundles, and thus exhibiting antibacterial properties [86]. Tomato shows global DNA hypomethylation after inoculation with root-parasitic nematodes [87]. DNA methylation is considered to regulate stress tolerance in plants. However, the dynamics of cytosine methylation in woody plants and its relevance to pathogenic responses are only partially understood. Mulberry (*Morus notabilis*) is an economically and ecologically important tree, the leaves of which are the main food for silkworms. Mulberry trees are often infected by various diseases, among which *Botrytis cinerea* is a primary pathogen. Xin *et al*. obtained whole-genome cytosine methylation profiles for mulberry using mock-treated leaves versus leaves inoculated with *B. cinerea* [73]. A decrease in mCG and mCHG levels and an increase in mCHH levels were observed in the inoculated samples compared with mock-treated samples. The cytosine methylation level of resistance-associated genes was decreased, whereas that of metabolism-associated genes was increased [73]. Silencing of the mulberry DNA methyltransferase *MnMET1* induced by Tobacco curculio virus improved resistance to *B. cinerea* in mulberry [73].

Among the biotic and abiotic stresses that affect potato (*Solanum tuberosum*), *Fusarium* dry rot is one of the most important diseases that significantly impacts tuber production, storage, and processing. Shi *et al*. conducted transcriptome and methylome analysis of potato tubers treated with different concentrations of deoxynivalenol (DON). The overall cytosine methylation level of DON-treated tubers was decreased notably compared with that of the control. Interestingly, the genes with differentially methylated regions were highly enriched in resistance-related metabolic pathways, which revealed that cytosine methylation plays a critical role in potato disease resistance and has excellent potential to improve biotic stress resistance [66]. Melon is an important cash crop worldwide that is severely damaged by powdery mildew. Understanding of the powdery mildew resistance mechanism in melon is still limited. It was found that the net photosynthetic rate (*P*_n_), stomatal conductance (*G*_s_), actual photochemical efficiency (ФPSII), and maximum PSII quantum efficiency (*F*_v_*/F*_m_) were significantly lower in susceptible melon compared with those of the control, by comparative transcription and methylome analysis [65]. Among the 4808 differentially expressed genes (DEGs) identified, 932 were associated with hypermethylation and 603 were associated with hypomethylation under *Podosphaera xanthii* stress, further identifying a set of genes associated with resistance. This finding revealed that DNA methylation is directly involved in the resistance of melon to *P. xanthii* infection, providing potential targets for future research on melon resistance to powdery mildew [65].

The methylation level of tobacco (*Nicotiana tabacum*) decreases significantly after inoculation with the Tobacco mosaic virus. Viral infection causes a significant increase in DNA methylation in tobacco, but hypomethylation occurs in the leucine repeat sequence region associated with disease resistance [88]. Viral infection leads to decreased methylation levels in hops [89]. It is clear from previous studies that pathogenic fungi and viruses infecting plants may cause changes in the overall cytosine methylation level of the plant. Furthermore, genes associated with disease resistance often show a decrease in cytosine methylation level, which activates or enhances gene expression and thus increases disease resistance.

### 5.2. Role of cytosine methylation in response to abiotic stress

Cytosine methylation dynamics also play an essential role in responses to abiotic stresses, which mainly include heavy metals, drought, high temperature, cold, and salt. Studying the effects of abiotic stresses on plant DNA methylation will help with understanding plant stress-tolerance mechanisms.

**Figure 2:**
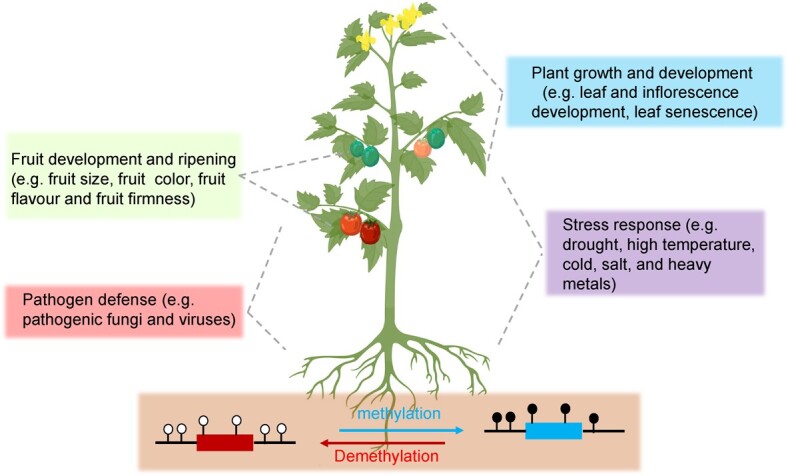
Roles of cytosine methylation in horticultural crops. DNA methylation is an essential epigenetic modification that maintains the dynamic balance of cytosine methylation in plants under the regulation of methyltransferase and demethylase. DNA methylation in plants may suppress the transcription of transposons and exogenous DNA to maintain genomic stability. Disruption of cytosine methylation in plants can lead to developmental abnormalities, such as in fruit development and ripening (e.g. fruit size, fruit color, fruit flavor, and fruit firmness), and plant growth and development (e.g. leaf and inflorescence development, and leaf senescence). Plants also can actively reduce stress damage by regulating their own regulatory mechanisms. Cytosine methylation dynamics play crucial roles in response to not only biotic stresses (e.g. pathogenic fungi and viruses), but also abiotic stresses (e.g. high temperature, drought, cold, salt, and heavy metals).

Drought is the environmental stress that causes the most damage to plant yield and growth rate. To cope with drought stress, plants have evolved complex mechanisms, including DNA methylation regulation. For example, Niu *et al*. reported that a high expression level of the drought-inducible *MdRFNR1* gene, which may increase the drought tolerance of apple, was positively correlated with the MIT-MdRF1 insertion and its cytosine methylation. The MdSUVH–MdDNAJ complex mediates the DNA methylation level under drought stress [90]. In addition, the grafted scion, an essential requirement for fruit trees in horticulture, is also associated with alternating DNA methylation dynamics under drought [91]. Cytosine-5-methyltransferases and demethylases play essential roles in maintaining the cytosine methylation status of the plant genome under drought stress [92, 93].

DNA methylation can enable plants to acquire tolerance to adversity. For example, a decrease in the methylation level of cold-responsive genes in the tea plant (*Camellia sinensis*) under low-temperature stress leads to an increase in the transcript level of cold-responsive genes, thus improving cold tolerance [72]. In addition, low temperature delays ripening and reduces spoilage of fruit in postharvest storage, and refrigeration reduces the loss of fruit flavor. The loss of flavor in tomato fruit is due to loss of flavor volatiles. For example, the contents of flavor-related volatiles were reduced in the fruit of two tomato cultivars after chilling, which was associated with significantly reduced transcript levels for key volatile synthases, namely *branched-chain aminotransferases* (*BCAT*s), *lipoxygenase* (*LoxC*), *ALCOHOL DEHYDROGENASE 2* (*ADH2*), *hydroperoxide lyase* (*HPL*), and *ALCOHOL ACETYLTRANSFERASE 1* (*AAT1*) [51]. Cytosine methylation in the promoter region increased after 7 days of refrigeration as detected by WGBS analysis and, in addition, the abundance of *DML2* transcripts was reduced during refrigeration, suggesting that the reduction in transcript levels of volatiles and maturation-related TFs is associated with changes in methylation levels [51].

Other stresses, including high temperature, salt, and heavy metal pollution of the soil, also cause a host of morphological, physiological, and biochemical changes that are severely harmful to plant growth and development. In most cases, the total methylation levels of heat-stressed plants are reduced, such as in strawberry and oilseed rape (*Brassica napus*) [94, 95]. In broccoli (*Brassica oleracea*), an elevated temperature (22 or 28°C) induces hypermethylation of the genome, leading to inhibition of the expression of distinctive genes highly expressed in the shoot apex and subsequently resulting in abnormal floral development [96]. DNA methylation dynamics change in plants under salt stress, but plant responses to salt stress differ. For example, the total cytosine methylation level of potato is decreased under salt stress [97], whereas hypermethylation is observed with the transcriptional regulation of chromatin modifier genes in the genome of *Bruguiera gymnorhiza* under high-salinity stress [98]. In recent decades, heavy metal pollution in the soil has become a major environmental concern. The cytosine methylation level of plants shows dynamic changes under heavy metal stress, but the trend of the change differs among plant species. Methylation levels decrease in *Trifolium repens* and *Cannabis sativa* under stress from heavy metals such as cadmium (Cd), nickel, and chromium (Cr). In contrast, *Brassica campestris* and *Raphanus sativus* show elevated cytosine methylation levels under aluminum, Cd, and Cr stresses [99]. These results suggest that cytosine methylation is involved in biotic and abiotic stress responses to balance plant development and stress tolerance.

## 6. Conclusions and perspectives

DNA methylation is an essential epigenetic modification that maintains the dynamic methylation balance in plants under the regulation of methylation and demethylation enzymes. In this review, we discuss recent studies on the regulatory mechanisms of cytosine methylation in the growth and development of horticultural crops, such as the effects of DNA methylation on maturation, senescence, plant height, and organ size (Fig. 2).

DNA methylation levels are significantly downregulated during tomato fruit ripening and treatment with DNA methylation inhibitors alters fruit ripening in several species [1, 12]. Additionally, plants are subject to biotic and abiotic stresses during growth and development, leading to changes in DNA methylation levels, which control the expression of resistance genes. Therefore, cytosine modification may be valuable for the improvement of horticultural crops. Natural epialleles responsible for important traits have been identified and isolated in various species, such as tomato. Engineered epialleles may provide an important source of phenotypic variation of utility in crop breeding. Moreover, CRISPR/dCas9 technology is now a readily available tool to enable scientists to modify the methylation status of the promoter of specific genes by flexible use of the methylase or demethylase domain, thereby achieving breakthroughs in phenotypic diversity by regulating gene expression [100].

The regulatory roles of cytosine methylation in horticultural crops are a fascinating topic of research. However, a number of important questions remain unanswered: the mechanisms underlying the silencing role of DNA demethylation and how promoter methylation activates gene transcription *in vivo*; the signals that trigger epigenetic changes during different processes; the mechanisms underlying the crosstalk among epigenetic modifications, plant hormones, and transcription regulators; and the mechanism through which epigenetic regulators are specifically recruited to loci in different cell types and genomic targets. The roles of cytosine methylation regulation in horticultural crops are a crucial and challenging area of biological research, with many unsolved mysteries.

## Acknowledgements

This work was supported by the National Natural Science Foundation of China (32270367), the Office of Education of Anhui Province for Distinguished Young Scholars (2022AH020061), and the National Key Research and Development Program of China (2021YFA1300401 and NK2022010301).

## Author contributions

P.L. and Q.N. prepared the review and wrote the original draft. R.L., Y.X., C.Z., Q.N., and Z.L. conducted the literature review and edited the manuscript.

## Conflict of interest

None declared.
